# Pacemaker and Defibrillator Implantation in Patients with Transposition of the Great Arteries

**DOI:** 10.19102/icrm.2017.080405

**Published:** 2017-04-15

**Authors:** Alex F. Grubb, Gautam Shah, Peter F. Aziz, Richard A. Krasuski

**Affiliations:** ^1^Cleveland Clinic Lerner College of Medicine of Case Western Reserve University, Cleveland, OH; ^2^Department of Internal Medicine, Cleveland Clinic Health System, Cleveland, OH; ^3^Department of Pediatric Cardiology, Cleveland Clinic Children’s Hospital, Cleveland, OH; ^4^Cardiology Division, Duke University Health System, Durham, NC

**Keywords:** Congenital heart disease, defibrillation, pacemaker, transposition of great arteries

## Abstract

Transposition of the great arteries (TGA) is represented in 5% to 7% of patients with congenital heart disease. These patients face a significant burden of arrhythmia and sudden cardiac death throughout their lives, and many eventually undergo pacemaker or cardiac-defibrillator implantation. Outcomes data following device implantation in this population, however, are limited. From an electrophysiologic database at a large, tertiary care medical center, we identified 63 TGA patients (34 with dextro (d)-TGA and 29 with levo (l)-TGA) with systemic right ventricles receiving an implantable cardiac device from 1996 to 2014. Medical records were reviewed for demographic, echocardiography and device interrogation data. Overall, l-TGA patients were older than d-TGA patients when they underwent initial device implantation (35.6 ± 18.2 versus 17.3 ± 10.6 years, p<0.001), and had more concomitant cardiac defects (55% versus 12%, p<0.001). Survival following initial device implantation was similar between l-TGA and d-TGA (72% versus 74%, p = 1.00), despite the baseline difference in age. Twenty-four patients underwent implantable cardioverter-defibrillator (ICD) implantation: 18 for primary intervention (11 l-TGA and seven d-TGA), and six for secondary prevention (four l-TGA and two d-TGA). Sixty-seven percent of patients in the secondary prevention group had appropriate shocks, compared with 0% of primary prevention patients. Patients with ICD discharge were more likely to have concomitant heart defects (100% versus 30%, p = 0.011). Despite being significantly younger, d-TGA patients had similar survival rates following device implant to l-TGA patients. Patients with TGA and sustained ventricular arrhythmias are at high risk for subsequent events, and typically benefit from ICD implantation. The role of prophylactic ICD implantation in this population, however, remains uncertain.

## Introduction

Transposition of the great arteries (TGA) represents about 5% to 7% of all congenital heart disease, and is characterized by ventriculo-arterial discordance, wherein the pulmonary arteries originate from the left ventricle and the aorta arises from the right ventricle.^1,2^ Based upon the atrioventricular concordance or discordance, the defect is further classified as dextro-transposition of the great arteries (d-TGA) or levo-transposition of the great arteries (l-TGA), respectively.^3^

Children born with d-TGA are generally cyanotic and often require an operative intervention for survival.^2^ Though arterial switch is now the standard of care for operative intervention, many patients in the current adult d-TGA population underwent Mustard and Senning (atrial baffle procedures) as children. In contrast, patients with l-TGA, particularly in the absence of associated lesions, may remain asymptomatic and progress into early and even later adulthood without the abnormality being recognized.^4^

Owing to the evolution of surgery in d-TGA and the progression into adulthood of patients with unidentified l-TGA, cardiologists now encounter an increasing number of adults with TGA.^4–6^ The most common problems affecting these patients long term include arrhythmias, heart failure, and sudden cardiac death (SCD).^3,7,8^ Because of these complications, a large number of TGA patients eventually require pacemakers or implantable cardioverter-defibrillators (ICDs). Evidence currently remains limited regarding the appropriate use of such devices in this patient population; despite that, appropriate use of ICDs is of particular interest given the high SCD risk. While use of ICDs for secondary prevention is generally accepted, implantation for primary prevention remains a topic of great scrutiny.

Although there appears to be sufficient evidence to justify ICD implantation in patients with symptomatic heart failure (NYHA functional class II and III) and/or ejection fraction less than 35%, no study to date has attempted to translate this benefit to adult congenital heart disease patients with comparable systemic ventricular dysfunction.^9,10^ Recent studies suggest benefits for ICDs in secondary prevention of SCD in cases of TGA.^7,11^ However, the use of ICDs for primary prevention in TGA remains uncertain.

In this study, we examined the clinical experiences of a single, large referral center, in an effort to further understand the arrhythmic substrates of l-TGA and atrially corrected d-TGA, including the indications for device implantation and subsequent clinical course and outcomes.

## Methods

After obtaining International Review Board approval for medical records research, we queried the Cleveland Clinic Heart and Vascular Institute Implantable Device Database for all patients with a diagnostic designation of “congenital heart disease” who underwent pacemaker or ICD implantation from 1996 to 2014. We then reviewed the electronic and paper medical records of each patient to identify TGA and specify the anatomic substrate. Diagnoses were further refined by reviewing the individual echocardiograms and any additional imaging results. Transposition patients with single-ventricle corrections (Fontan) or those with systemic left ventricles following Rastelli, Senning/Rastelli, or arterial switch procedures were excluded. Demographic data were collected for all patients, as were medical comorbidities and echocardio-graphic and electrophysiologic data. We deemed each patient’s device that had been initially recorded into the Cleveland Clinic Heart and Vascular Institute Implantable Device Database as the patient’s “index device.” Many of these patients, however, had had devices implanted prior to being seen for the first time at the Cleveland Clinic. Through a detailed review of the recorded history, we determined the first device ever implanted into these patients, their age at its implantation, and the total number of previous devices that the patient had gone through before presenting to our institution. We deemed the first device ever implanted into these patients as the “initial devices.”

Heart failure was defined by signs and/or clinical symptoms, and the index diagnosis was the first time that a Cleveland Clinic physician recorded this information within the patient’s problem list. All device interrogations conducted during follow-up were individually reviewed, and their data tabulated. “Appropriate shocks” were defined as therapies delivered to terminate sustained ventricular tachycardia (VT) and/or ventricular fibrillation (VF). Inappropriate shocks were defined as therapies delivered for any other reason. We confirmed all deaths using the social security death index. Event-free survival was defined as the time from implantation of initial device until either death or cardiac transplantation. Elevated defibrillation thresholds (DFTs) were defined as those greater than 25 J.

Data are presented as mean ± standard deviation for continuous variables, and as percentages for discrete variables. A comparison of dichotomous variables was performed using the chi-squared test or Fisher’s exact test whenever appropriate. Comparisons of continuous variables between the groups were performed using two-sided t-tests and one-way analysis of variance. The means of non-parametric data were compared using the Mann-Whitney U (Wilcox ranked sums) test. Statistical significance was assumed for p<0.05. Survival analyses were performed using the Kaplan-Meier method. Statistically significant differences in the survival functions were assessed with the Wilcoxon test. All analyses were performed using JMP version 12.0.1 (SAS Institute, Cary, NC).

## Results

We identified and confirmed 71 patients with TGA, including 38 patients with d-TGA and 33 patients with l-TGA, who received an implantable cardiac device between January 1996 and June 2014. Four patients with d-TGA and four patients with l-TGA were excluded due to undergoing previous arterial switch or Fontan or Rastelli repairs, thus yielding a final cohort of 34 d-TGA and 29 l-TGA patients with systemic right ventricles. The two groups were similar in terms of sex, the proportion of patients who had had device(s) prior to the index device, the average number of prior devices, the proportion of leads placed epicardially, the proportion who received an ICD as their initial device, and the percentage with a presence of heart failure at the time of index device implantation **(Table 1)**. l-TGA patients were significantly more likely to have additional cardiac defects than their d-TGA counterparts (55% versus 12%, p< 0.001). They were also considerably older at the time of initial device implant (35.6 ± 18.2 versus 17.3 ± 10.6 years, p< 0.001).

**Figure 1:**
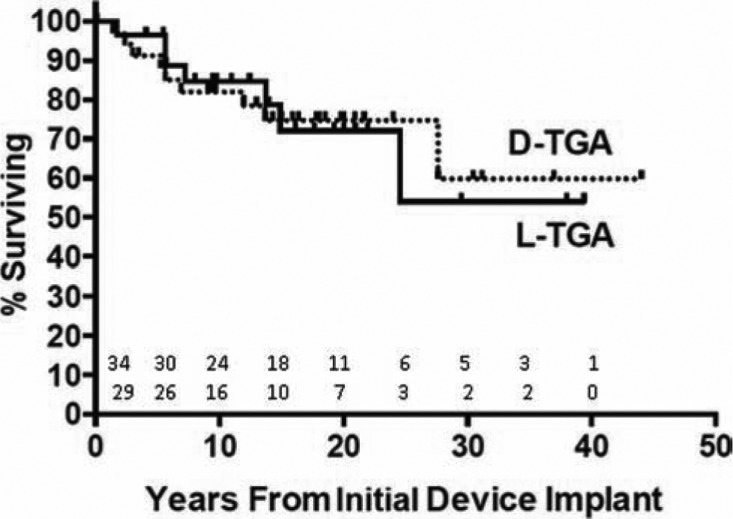
Kaplan-Meier curves comparing survival following implantation of initial cardiac device. No difference was seen between the dextro-transposition of the great arteries (d-TGA) and l-TGA (levo-transposition of the great arteries) groups (p = 0.998). l-TGA patients were, however, significantly older at the time of implant than their d-TGA counterparts (35.6 ± 18.2 versus 17.3 ± 10.6 years, p<0.001).

As expected, because of the nature of the defect, patients with d-TGA were significantly younger at the time of surgical repair (1.6 ± 2.1 versus 24.9 ± 20.4 years, p< 0.001), and also had a longer interval from their operative intervention until implantation of the initial device (16.6 ± 10.8 versus 7.5 ± 12.9 years, p = 0.016). Furthermore, more d-TGA patients received their initial device because of sinus node dysfunction (65% versus 10%, p< 0.001), while l-TGA patients were significantly more likely to receive an initial device due to atrioventricular (AV) block (12% versus 69%, p<0.001).

l-TGA patients had similar lengths of follow-up after implantation of the index device (6.4 ± 5.4 versus 4.4 ± 4.3 years, p = 0.108). Cardiac-related hospitalizations occurred in a similar fraction of patients in each group (77% of l-TGA and 61% of d-TGA patients, p = 0.24), with similar numbers of hospitalizations per patient (3.2 ±2.2 versus 3.4 ± 3.7, p = 0.85). Additionally, similar proportions of d-TGA and l-TGA patients went on to develop heart failure after implantation of their initial device (35% versus 44%, p = 0.47), with similar lengths of time to initial clinical diagnosis of heart failure (5.0 ± 6.8 versus 3.5 ± 4.6 years, p = 0.55). Finally, death or cardiac transplantation occurred at the same frequency during follow-up following initial device implantation in both groups (26% in d-TGA versus 28% in l-TGA, p = 1.00) **(Figure 1)**.

## ICD subanalysis

**Figure 2:**
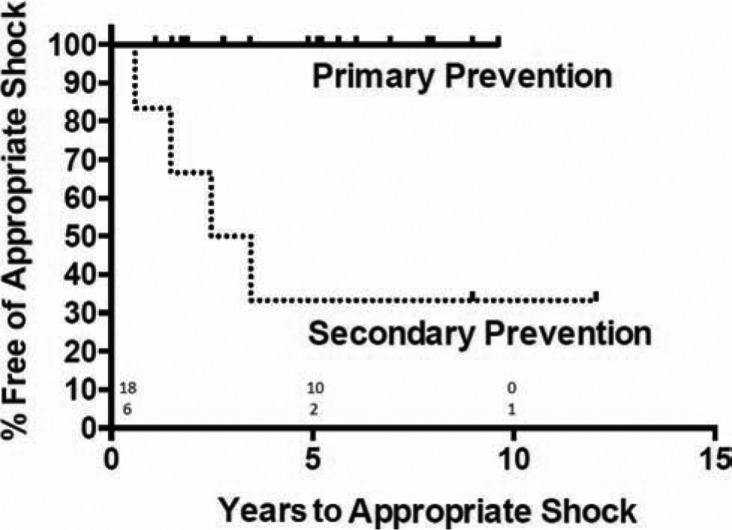
Time to appropriate shock following implantable cardioverter-defibrillators implantation. Median time to first shock for secondary prevention was 3.0 years (no events occurred in primary prevention arm), p = 0.002.

Fifteen patients (25%) had an ICD implanted as their initial device (nine l-TGA and six d-TGA). Of the 48 patients with a pacemaker as their initial device, eight were later upgraded to an ICD for primary prevention of sudden death (six l-TGA and two d-TGA), and one patient (one l-TGA) experienced sustained ventricular arrhythmia, necessitating an upgrade to an ICD. Of the 24 patients who eventually underwent ICD implantation, 18 received a device for primary prevention (11 l-TGA and seven d-TGA), and six were implanted for secondary prevention (four l-TGA and two d-TGA). Both the primary and secondary prevention groups had similar distributions in terms of type of TGA and age at implantation of their first device **(Table 3)**. Although not significant, those in the secondary prevention groups trended towards being male (100% versus 50%, p = 0.052), having a lower BMI (24.5 ± 3.43 versus 28.0 ± 3.6, p = 0.054), and having additional cardiac defects (83% versus 39%, p = 0.155). Of the patients receiving ICDs for primary prevention, 15 were implanted due to the development of heart failure in the context of systemic ventricular dysfunction, two were implanted for non-sustained VT, and one was implanted for non-clinical sustained VT on electrophysiologic study. Device interrogation follow-up data spanned 139 patient-years (89 patient-years for primary prevention and 50 patient-years for secondary prevention). Only one patient received ICD discharges in the primary prevention group, and both therapies were inappropriate. In the secondary prevention group, 67% of patients had appropriate ICD discharge (0.34 shocks per patient-year, p< 0.001, compared with primary prevention) **(Figure 2)**. Each of these four therapies were in response to VF, and in three of these cases, the patients went on to have additional appropriate therapies at later dates.

There was only one instance of failed shock. This was in a d-TGA patient with a coarctation, who experienced a run of VT that progressed to VF. Initial antitachycardic pacing and a 23-J shock were unsuccessful; however, a subsequent 32-J shock was successful. Overall, patients with ICD discharge were similar in age at ICD implantation (46.7 ± 10.3 versus 42.8 ± 15.8 years, p = 0.561), and were more likely to have concomitant heart defects than were patients without ICD discharge (100% versus 40%, p = 0.047) **(Tables 3 and 4)**. None of the patients in either primary or secondary prevention groups had VT ablations. Ninety-one percent of patients had non-elevated DFTs **(Table 5)**. Both d-TGA and l-TGA had similar distributions of excellent (<10 J), good (11–15 J), or acceptable (16 J to 25 J) DFTs **(Table 5)**. Only two patients (8.75) had elevated DFTs. One, an l-TGA patient with a complex anatomy, had an abdominal ICD, epicardial leads, a history of atrial dysrhythmias, a QRS of 142 ms, and was on mexiletine and dofetilide. The other, a d-TGA patient, had severe systolic and AV dysfunction, and a QRS of 190 ms.

## Discussion

In a single institutional cohort of patients with transposition of the great arteries undergoing device implantation, we identified several expected and some unexpected differences between d-TGA repaired with atrial switch, and l-TGA. As expected, patients with d-TGA predominantly received pacemakers for sinus node dysfunction, while l-TGA patients received pacemakers for AV block. Accordingly, d-TGA patients were more frequently atrially paced. Following Senning or Mustard repair, d-TGA patients are at high risk for developing sinus node dysfunction, while l-TGA patients are at a greater risk for developing AV block, particularly following surgical or catheter-based procedures.^12–18^

Initial device implantation occurred at a much earlier age in individuals with d-TGA. It is likely that the earlier need for surgical intervention contributed to this finding. While d-TGA requires surgery to survive, in the absence of associated abnormalities, l-TGA can remain asymptomatic and undetected until late in life. Up to 90% of l-TGA patients, however, have additional cardiac anomalies, including ventricular septal defects, pulmonary outflow track obstruction, and tricuspid valve abnormalities.^19,20^ Our observed frequency of additional congenital defects in l-TGA (55%) was lower than what has previously been reported (∽90%). This may contribute to the difference in age at initial device implantation. Since lone l-TGA patients are frequently asymptomatic until later in life, there is less of a need for early surgical or catheter-based interventions that could precipitate heart block.^21^ Interestingly, despite the older age of l-TGA patients at initial device implantation, both groups developed heart failure at similar frequencies, and experienced similar rates of cardiovascular hospitalization. Despite sharing similar anatomic substrates (systemic right ventricles and systemic tricuspid valves), the significantly younger d-TGA patients had similar survival following device implantation to that of the older l-TGA patient population. In fact, both populations experienced considerable morbidity and a ∽ 4% yearly mortality, emphasizing the progressive nature of their myocardial disorder and the important role of close clinical follow-up completed by appropriately trained physicians.

Our results also highlight the need for a better understanding of which patients benefit from the use of ICDs for primary prevention. Of the 18 patients who received an ICD for primary prevention, not one of them received an appropriate shock, while one received inappropriate shocks for atrial dysrhythmia. On the other hand, two-thirds of the patients implanted for secondary prevention received appropriate shocks. These results are similar to the trends observed in a previously published multi-center registry, in which annual rates of appropriate shocks in atrially-corrected d-TGA patients were 0.5% in the primary prevention and 6% in the secondary prevention cohorts, respectively.^7^

Our study found elevated DFTs in 8.7% of patients with systemic right ventricles, similar to the 6% to 12% rates of elevated DFTs in the non-congenital population.^22–24^ Few data exist on DFTs in patients with systemic right ventricles. Some studies have suggested that suboptimal defibrillation should be anticipated within the d-TGA post-Mustard population.^25^ Many studies have found associations with factors such as QRS duration, antiar-rhythmic use, atrial fibrillation, and heart size with elevated DFT, yet no validated predictive model currently exists.^26–29^ It is likely that the elevated DFTs in our study were influenced by many of these same factors, which affect the non-congenital population. Still, care should be taken in these patients. Systemic right ventricles, depending on their position, can be troublesome, particularly if the right ventricle is significantly dilated, as they often are.

Unfortunately, many of the data that guide recommendations in this patient population are applied from the clinical trials of patients without congenital heart disease. Furthermore, many of these recommendations are derived from consensus; that is, expert opinion, rather than outcomes data.^30^ Inappropriate device implantation not only increases healthcare costs, but also potentially places the patient at additional clinical risk. Placing wires through atrial baffles in patients with d-TGA may incur hemodynamic consequences and increase the risk of infection, and potentially stroke, in the presence of baffle leak. Epicardially placed leads incur the additional procedural risk of necessitating the use of general anesthesia. Additionally, inappropriate shocks may result in physical pain, emotional stress and the development of anxiety, and even agoraphobia.^31,32^ Our data suggest that implanting an ICD for primary prevention in patients with TGA using the guidelines established in non-congenital populations may not provide benefit. Among patients with systemic right ventricles, some measure of systolic dysfunction is to be expected. By middle adulthood, it is estimated that over half of l-TGA patients, and nearly all d-TGA patients who underwent a Mustard or Senning procedure, will experience systolic dysfunction.^33,34^ Furthermore, non-sustained VT is fairly common in the setting of right ventricle dysfunction. As such, careful consideration should be given prior to reflexively implanting ICDs in this population, as the risks associated with such complex and technically challenging procedures may outweigh the potential benefits. Clearly, this is an area that is in dire need of additional study.

This study has significant limitations. Owing to the retrospective nature of the data collection, the precise cause of death was not available for the majority of the TGA patients who had died. It would have been of interest to know which patients died from cardiac and arrhythmic causes and their unique clinical characteristics. Additionally, the retrospective study design limits the study’s source of data to only that which was recorded in the electronic and paper medical records within our hospital system. The differential availability of data within the record, different lengths of follow-up, and differing frequencies of follow-up also limited the types of variables we could accurately and reliably compare. These problems frequently plague studies in adults with congenital heart disease. Future studies should compare different pacing modes and their frequencies, incidence of atrial tachyarrythmias, and the frequency and utility of anti-tachycardia pacing between these two groups. Lastly, the small number of study participants does somewhat limit overall generalizability. Owing to the generalized lack of data with which to guide treatments in these patients, however, this study’s data should still be taken into account when treating TGA patients with pacemakers or ICDs, and in designing future studies.

Overall, this study supports the implantation of ICDs for secondary prevention in patients with systemic right ventricles, especially in those patients who have more complex congenital heart disease. Owing to the small number of study participants, however, future prospective data are needed in order to confirm these findings and more clearly delineate the congenital heart disease patients who benefit most from ICD implantation.

## Figures and Tables

**Table 1:** Population Demographics

		Total (n = 63)	d-TGA (n = 34)	I-TGA (n = 29)	p
Age at Initial Device	Mean ± SD	25.9 ± 17.2	17.3 + 10.6	35.6 ± 18.5	<0.001
Implantation	Median (IQR)	22.2 (14.6–35.8)	16.2 (9.3–22.2)	35.1 (23.3–50.2)	
Male		68%	74%	62%	0.418
Had Previous Device		63%	65%	60%	0.793
Number of Prior Devices	Mean ± SD	1.6 ± 0.8	1.7 ± 0.8	1.5 ± 0.9	0.226
ICD (as Initial Device)		24%	15%	34%	0.235
Lead Type	Epicardial	14%	12%	17%	0.721
	Endocardial	86%	88%	83%	
Index Device Type	Single-chamber	16%	18%	10%	0.009
	Dual-chamber	70%	82%	55%	
	Bi-ventricular	14%	0%	34%	
Concomitant Defects		32%	12%	55%	<0.001
≥ Moderate Systemic		58%	62%	56%	0.782
Ventricular Dysfunction					
≥ Moderate Systemic AV		41%	24%	58%	0.0209
Valve Regurgitation					

Data are presented as %, unless otherwise stated. AV: atrioventricular; d-TGA: dextro-transposition of the great arteries; ICD: implantable cardioverter-defibrillator; IQR: interquartile range; l-TGA: levo-transposition of the great arteries; SD: standard deviation.

**Table 2:** Types of Additional Defects

Defect Type	d-TGA	l-TGA
Coarctation	2	1
Ventricular Septal Defect	1	13
Pulmonic Stenosis	1	6
Ebsteins	0	1
Pulmonary Atresia	0	1
Atrial Septal Defect	0	1
Dextrocardia	0	3
Bicuspid Aortic Valve	0	1
Dysplastic Tricuspid Valve	0	1
Complete Congenital Heart Block	0	1

**Table 3:** ICD Cohort Demographics

		Primary Prevention ICD	Secondary Prevention ICD	Pacemaker-only Cohort	P
Type of Defect	d-TGA	33%	33%	67%	1.00
	l-TGA	67%	67%	33%	
Concomitant Defects		39%	83%	21%	0.1550
Male		50%	100%	72%	0.052
Age at First Device Implantation (Mean ± SD)		36.0 ± 18.0	40.5 ± 9.8	18.3 ± 13.4	0.424
Death or Transplant		22%	50%	26%	0.307
≥ Moderate Systemic Ventricular Dysfunction		83%	50%	48%	0.307
≥ Moderate Systemic AV Valve Regurgitation		71%	33%	23%	0.162

Data are represented in %, unless otherwise stated. ICD: implantable cardioverter-defibrillator; SD: standard deviation; TGA: transposition of the great arteries. P-values represent comparison between primary and secondary prevention. The pacemaker- only group is included for reference.

**Table 4:** ICD Outcomes

	Patient- years	Percentage with Appropriate Shocks	Number of Appropriate Shocks	Percentage with Inappropriate Shocks	Number of Inappropriate	Time to First Appropriate	Time to First Inappropriate	Incident Event Rate
Primary (n = 18)	89	0%	0	6%	1	–	4.1 years	0
Secondary (n = 6)	50	67%	17	0%	0	3.0 years	–	120/1,000 patient-years

Patient-years: patient-years of follow-up.

**Table 5:** Defibrillation Thresholds

	Total (n = 23)	d-TGA (n = 8)	l-TGA (n = 15)
<10 J	52%	63%	47%
11–15 J	13%	13%	13%
15–25 J	26%	13%	33%
25 + J	9%	13%	7%

## References

[1] Digilio MC, Casey B, Toscano A, Calabro R, Pacileo G, Marasini M (2001). Complete transposition of the great arteries: patterns of congenital heart disease in familial precurrence. Circulation..

[2] Brickner ME, Hillis LD, Lange RA (2000). Congenital heart disease in adults. Second of two parts. N Engl J Med..

[3] Warnes CA (2006). Transposition of the great arteries. Circulation..

[4] Beauchesne LM, Warnes CA, Connolly HM, Ammash NM, Tajik J, Danielson G (2002). Outcome of the unoperated adult who presents with congenitally corrected transposition of the great arteries. J Am Coll Cardiol..

[5] Puley G, Siu S, Connelly M, Harrison D, Webb G, Williams WG (1999). Arrhythmia and survival in patients >18 years of age after the Mustard procedure for complete transposition of the great arteries. Am J Cardiol..

[6] Helbing WA, Hansen B, Ottenkamp J, Rohmer J, Chin JG, Brom AG (1994). Long-term results of atrial correction for transposition of the great arteries. Comparison of Mustard and Senning operations. J Thorac Cardiovasc Surg..

[7] Khairy P, Harris L, Landzberg MJ, Fernandes SM, Barlow A, Mercier LA (2008). Sudden death and defibrillators in transposition of the great arteries with intra-atrial baffles: a multicenter study. Circ Arrhythm Electrophysiol..

[8] Sodhi SS, Cedars AM (2015). Primary prevention of sudden cardiac death in adults with transposition of the great arteries: A review of implantable cardioverter-defibrillator placement. Tex Heart Inst J..

[9] Moss AJ, Zareba W, Hall WJ, Klein H, Wilber DJ, Cannom DS (2002). Prophylactic implantation of a defibrillator in patients with myocardial infarction and reduced ejection fraction. N Engl J Med..

[10] Bardy GH, Lee KL, Mark DB, Poole JE, Packer DL, Boineau R (2005). Amiodarone or an implantable cardioverter-defibrillator for congestive heart failure. N Engl J Med..

[11] Khanna AD, Warnes CA, Phillips SD, Lin G, Brady PA (2011). Single-center experience with implantable cardioverter-defibrillators in adults with complex congenital heart disease. Am J Cardiol..

[12] Flinn CJ, Wolff GS, Dick M, Campbell RM, Borkat G, Casta A (1984). Cardiac rhythm after the Mustard operation for complete transposition of the great arteries. N Engl J Med..

[13] Manning PB, Mayer JE, Wernovsky G, Fishberger SB, Walsh EP (1996). Staged operation to Fontan increases the incidence of sinoatrial node dysfunction. J Thorac Cardiovasc Surg..

[14] Ghai A, Harris L, Harrison DA, Webb GD, Siu SC (2001). Outcomes of late atrial tachyarrhythmias in adults after the Fontan operation. J Am Coll Cardiol..

[15] Walsh EP, Cecchin F (2007). Arrhythmias in adult patients with congenital heart disease. Circulation..

[16] Huhta JC, Maloney JD, Ritter DG, Ilstrup DM, Feldt RH (1983). Complete atrioventricular block in patients with atrioventricular discordance. Circulation..

[17] Connelly MS, Liu PP, Williams WG, Webb GD, Robertson P, McLaughlin PR (1996). Congenitally corrected transposition of the great arteries in the adult: functional status and complications. J Am Coll Cardiol..

[18] Weindling SN, Saul JP, Gamble WJ, Mayer JE, Wessel D, Walsh EP (1998). Duration of complete atrioventricular block after congenital heart disease surgery. Am J Cardiol..

[19] Graham TP, Bernard YD, Mellen BG, Celermajer D, Baumgartner H, Cetta F (2000). Long-term outcome in congenitally corrected transposition of the great arteries: a multi-institutional study. J Am Coll Cardiol..

[20] Presbitero P, Somerville J, Rabajoli F, Stone S, Conte MR (1995). Corrected transposition of the great arteries without associated defects in adult patients: clinical profile and follow up. Br Heart J..

[21] Hornung TS, Calder L (2010). Congenitally corrected transposition of the great arteries. Heart..

[22] Mainigi SK, Cooper JM, Russo AM, Nayak HM, Lin D, Dixit S (2006). Elevated defibrillation thresholds in patients undergoing biventricular defibrillator implantation: incidence and predictors. Heart Rhythm..

[23] Russo AM, Sauer W, Gerstenfeld EP, Hsia HH, Lin D, Cooper JM (2005). Defibrillation threshold testing: is it really necessary at the time of implantable cardioverter-defibrillator insertion?. Heart Rhythm..

[24] Shukla HH, Flaker GC, Jayam V, Roberts D (2003). High defibrillation thresholds in transvenous biphasic implantable defibrillators: clinical predictors and prognostic implications. Pacing Clin Electrophysiol..

[25] Michael KA, Veldtman GR, Paisey JR, Yue AM, Robinson S, Allen S (2007). Cardiac defibrillation therapy for at risk patients with systemic right ventricular dysfunction secondary to atrial redirection surgery for dextro-transposition of the great arteries. Europace..

[26] Nagai T, Kurita T, Satomi K, Noda T, Okamura H, Shimizu W (2009). QRS prolongation is associated with high defibrillation thresholds during cardioverter-defibrillator implantations in patients with hypertrophic cardiomyopathy. Circ J..

[27] Raitt MH, Johnson G, Dolack GL, Poole JE, Kudenchuk PJ, Bardy GH (1995). Clinical predictors of the defibrillation threshold with the unipolar implantable defibrillation system. J Am Coll Cardiol..

[28] Ujhelyi MR, Schur M, Frede T, Bottorff MB, Gabel M, Markel ML (1996). Mechanism of antiarrhythmic drug-induced changes in defibrillation threshold: role of potassium and sodium channel conductance. J Am Coll Cardiol..

[29] Mizukami K, Yokoshiki H, Mitsuyama H, Watanabe M, Tenma T, Matsui Y (2014). Predictors of high defibrillation threshold in patients with implantable cardioverter-defibrillator using a transvenous dual-coil lead. Circ J..

[30] Epstein AE, DiMarco JP, Ellenbogen KA, Estes NA, Freed-man RA, Gettes LS (2008). ACC/AHA/HRS 2008 Guidelines for Device-Based Therapy of Cardiac Rhythm Abnormalities: a report of the American College of Cardiology/American Heart Association Task Force on Practice Guidelines (Writing Committee to Revise the ACC/AHA/NASPE 2002 Guideline Update for Implantation of Cardiac Pacemakers and Antiarrhythmia Devices) developed in collaboration with the American Association for Thoracic Surgery and Society of Thoracic Surgeons. J Am Coll Cardiol..

[31] Sears SF, Conti JB (2002). Quality of life and psychological functioning of ICD patients. Heart..

[32] Vlay SC, Olson LC, Fricchione GL, Friedman R (1989). Anxiety and anger in patients with ventricular tachyarrhythmias. Responses after automatic internal cardioverter defibrillator implantation. Pacing Clin Electrophysiol..

[33] Carrel T, Pfammatter JP (2000). Complete transposition of the great arteries: surgical concepts for patients with systemic right ventricular failure following intraatrial repair. Thorac Cardiovasc Surg..

[34] Hörer J, Herrmann F, Schreiber C, Hermann F, Schreiber C, Cleuzio J (2007). How well are patients doing up to 30 years after a mustard operation?. Thorac Cardiovasc Surg..

